# Analysis of SnO_2_|WO_3_ Heterocontact Properties during the Detection of Hydrogen Sulphide

**DOI:** 10.3390/s141120480

**Published:** 2014-10-30

**Authors:** Patrycja Suchorska-Woźniak, Olga Rac, Marta Fiedot, Helena Teterycz

**Affiliations:** Wroclaw University of Technology, Faculty of Microsystem Electronics and Photonics, Janiszewskiego 11/17, Wroclaw 50-372, Poland; E-Mails: olga.rac@pwr.edu.pl (O.R.); marta.fiedot@pwr.edu.pl (M.F.); helena.teterycz@pwr.edu.pl (H.T.)

**Keywords:** metal oxide, gas sensors, hydrogen sulphide, heterocontact

## Abstract

The main objective of the paper was the analysis of the properties of SnO_2_|WO_3_ heterocontact as well as the determination of its response to 50 ppm of hydrogen sulphide. It was noticed that the sensitivity of the sensor being tested to hydrogen sulphide depended significantly on the polarization direction. When its parameters were compared with those of sensors the gas-sensitive layer of which was made only from one type of metal oxide, a high impact of the heterocontact on the electric charge transport was noticed. The value of the activation energy of the electric conductivity is clearly dependent on the polarization direction. A mechanism of physicochemical processes occurring in a planar sensor with a heterocontact was suggested, and three areas differing in the width of depleted layer, where the inter-phase boundary of SnO_2_|WO_3_ had the most essential impact on the parameters of the sensor being tested, were distinguished and described.

## 1. Introduction

Semiconductive gas sensors are cheap, compact and their resistance depends on the composition of gaseous atmosphere. Individual constituents of the gaseous atmosphere participate in chemical reactions occurring on the surface of grains of the gas-sensitive material. Those sensors have been manufactured for several dozens of years, although their usage parameters are still at an unsatisfactory level. In order to improve these parameters, especially their selectivity, new sensing materials are being sought [1], their construction is being modified [2], as well as various measuring methods are used [3]. Resistive gas sensors usually comprise a single-phase gas-sensitive material, however, sensors in which the gas-sensitive layer is a multi-phase material are also manufactured. The sensors that contain a multi-phase material are characterized by a better selectivity than those containing a single-phase material [4–7]. Heterocontacts are formed between grains of varied chemical composition in gas-sensitive multi-phase materials. Similar heterocontacts are formed when sensors are constructed of two different layers [8–10].

The presence of a heterocontact between the grains of a sensor material or between the sensor layers modifies the sensor parameters. Gases are adsorbed not only on the surface of grains having defined physicochemical properties but in the connection area as well. For this reason the analysis of the phenomena occurring in this type of systems is very difficult. Yet, it is required in order to be able to fully use the presence of a heterocontact to improve the useful parameters of chemical sensors. Usually the sensors that contain heterocontacts are made from two mechanically inter-connected ceramic metals [8,11]. However, such a solution of the structure design does not ensure a stable connection.

This article presents the structure design of a planar sensor made using the screen print technology in which a gas-sensitive material of one type was printed on one measuring electrode and a gas-sensitive material of another type was printed on another measuring electrode. A heterocontact was formed between these layers. The sensors having such a structure design were characterized by a varying sensitivity depending on the polarization direction. As gas-sensitive materials two non-doped oxides were used: tin dioxide and tungsten trioxide. WO_3_ was chosen because it differs from SnO_2_ not only with its crystalline structure, but also in its electrical and gas-sensitive properties [12]. In addition, the sensors having this structure and composition demonstrate different sensitivity to hydrogen sulphide, depending on the polarization direction. So far, such results together with a rigorous analysis of the phenomena have not been published.

## 2. Experimental Section

**Figure 1.**
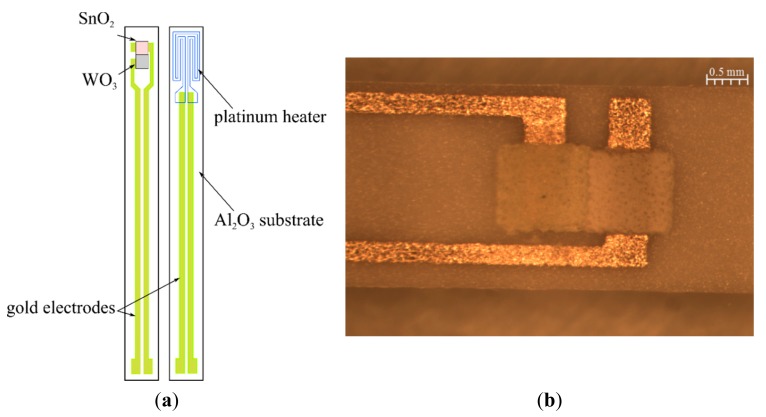
(**a**) Schematic diagram of a sensor; (**b**) Ready-made resistance sensor with a heterocontact.

A sensor was made on the alundum ceramics (96% Al_2_O_3_) of a 250 μm thickness. The platinum heater, having the shape of a meander and golden conducting paths (electrical leads to the platinum meander), were made on one side on the alundum ceramics (Figure 1). The dimensions of a single structure of the sensor are 25.40 × 2.45 mm^2^ but the area of the platinum meander totals *ca*. 7.10 mm^2^.

Golden electrodes were made on the other side of the substrate, on which two different sensor materials of a 40 μm thickness each were printed. The spacing between the electrodes was *ca*. 500 μm. Tin dioxide was printed on one electrode and tungsten trioxide was printed on another one. Sensor layers were printed in such a way so that they had a direct contact to each other, and they formed a heterocontact presented in Figure 1b. Such a design structure of the sensor allowed us to test the impact of the phenomena occurring within the heteroconnector area on the output signal of the sensor.

The metal oxide powders designed for the construction of the sensor were prepared by authors. The SnO_2_ synthesis carried out using the modified Okazaki's method [13]. As a precursor, a solution of anhydrous tin chloride (IV), glacial acetic acid and isopropanol were used. This solution was added drop-wise to deionized water at a temperature of 80 °C. As a result of the precipitation reaction hydrated tin dioxide and hydrochloric acid was formed according to the Equation (1):

(1) $$SnCl_{4}+xH_{2}O\underset{\to }{80{}^{\circ}c}SnO_{2}\cdot (x-2)H_{2}O+4HCl$$

Chlorine ions were removed from the suspension during the filtration of the precipitate, using a water solution of isopropyl alcohol. Afterwards, the SnO_2_ precipitate was dried in the lyophilisation process. Tungsten trioxide was obtained in the process of a thermal decomposition of hydrated tungstic-silicic acid (H_8_[Si(W_2_O_7_)_6_]·nH_2_O). The thermal decomposition was run at 650 °C during 5 h.

The ESL-403 thinner made by ESL Europe (Reading, England) was used for the preparation of pastes. The paste of a given material was printed twice, through drying the layers after each screen print, first at room temperature, next at 125 °C, during 10 min. A complete structure of the sensor was fired at 850 °C during 2 h.

The crystal structure of the powders was determined using a Philips Materials Research Diffractometer (Philips, Mahwah, NJ, USA) provided with a source of Cu Kα X-ray radiation. XRD measurements were made within the range of angle values of Θ/2Θ. The microstructure of the applied materials was tested using a scanning microscope type JSM 5800 LV made by Jeol (Tokyo, Japan).

**Figure 2.**
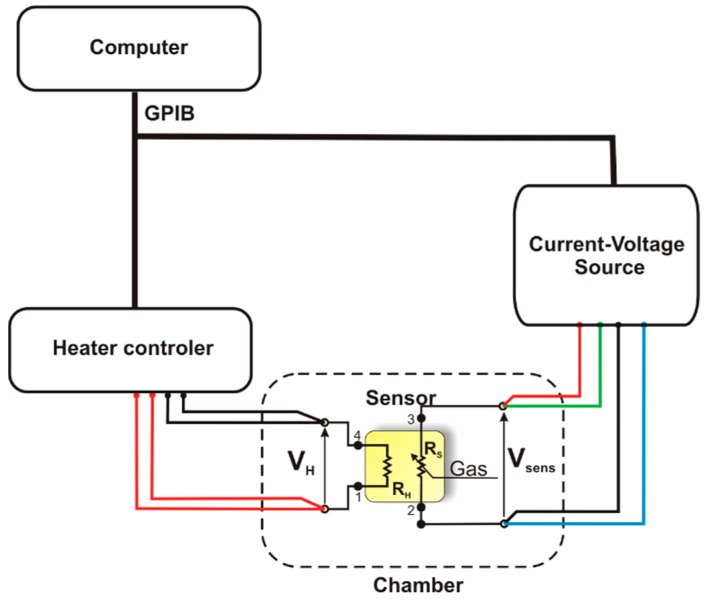
Schematic diagram of the construction of a measuring test stand used during the characterisation of sensors using the Temperature-Stimulated Conductance method and the techniques of voltammetry.

The kinetics of physicochemical processes occurring on the surface of gas-sensitive materials strongly depends on temperature, therefore the characterisation of sensors was made using a method called Temperature Stimulated Conductance (TSC) and electro-chemical methods. During the measurements using the TSC method, the sensor being tested was placed in a measurement chamber containing a gaseous atmosphere having a precisely defined composition. The operation temperature of the heater was changed linearly in cycles, with a constant rate of 2 deg/s within the range of 150 °C to 750 °C. The value of intensity of the electric current flowing through a gas-sensitive material was recorded during the temperature increase and temperature decrease. The gas-sensitive structure was polarized with a constant voltage using a Keithley 2400 current-voltage source (Keithley Instruments Inc., Cleveland, OH, USA), and the value of electric current was measured. Afterwards, using the Ohm's law, the resistance and conductance of the gas-sensitive material was determined. As illustrated in Figure 2, electric measurements were carried out at a test stand comprising a HP E3632 A power supply unit (Agilent Technologies Inc., Santa Clara, CA, USA) type, a Keithley 2400 current -voltage source, the measurement chamber and a PC provided with appropriate software.

Current-voltage characteristics of sensors at various temperatures were recorded using an potentiostat-galvanostat type SI 1287 from Solartron Analytical (Farnborough, England), with the use of the CorrWare software from Scribner Associates Inc. (Southern Pines, NC, USA). During the measurements, the value of the electrode polarization voltage was changed in cycles and linearly and, at the same time, the measurements of electric current intensity were performed.

The electric testing was made in synthetic air of a constant humidity (30% RH) and in air of the same humidity containing hydrogen sulphide (50 ppm). The temperature characteristics of TS-conductance of the sensors were determined on the basis of temperature and current changes results.

## 3. Results and Discussion

**Figure 3.**
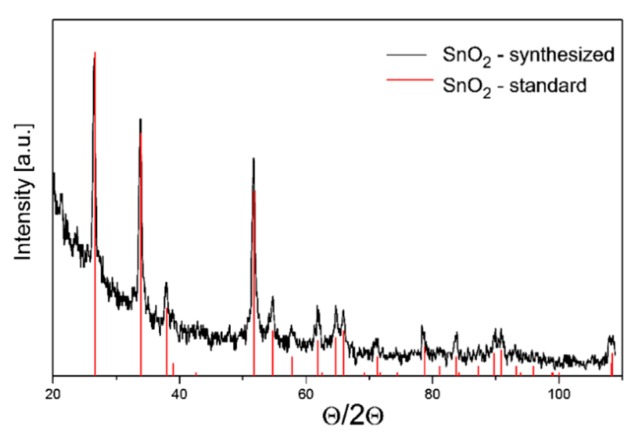
X-ray diffractograms of the standard SnO_2_ and the powder SnO_2_ obtained by the authors.

**Figure 4.**
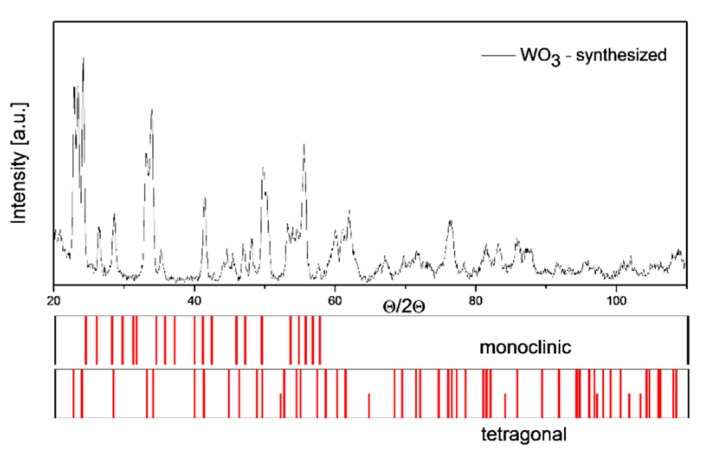
X-ray diffractograms of the powder WO_3_ obtained in the thermal decomposition process of the tungstic-silicic acid.

The testing carried out using the X-ray diffraction method presented in Figure 3, revealed that tin dioxide powder has typical crystallographic structure, *i.e.*, rutile type. On the other hand, as the XRD results revealed the tungsten (VI) oxide powder is a multi-phase material that contains both crystallites of a monoclinic and tetragonal phase (Figure 4).

Based on the analysis of the half width of peaks appearing on the diffractograms, using Scherrer formula described by the Equation (2), the average size of crystallites was determined:

(2) $$D=\frac{K_{\beta }\cdot \lambda }{\beta_{½}\cdot \text{cos}\theta }$$

where K_β_—the shape factor amounting to *ca*. 0.9 for particles of a spherical shape, λ—the X-ray radiation, β—the full width half maximum, FWHM, of the peak.

**Figure 5.**
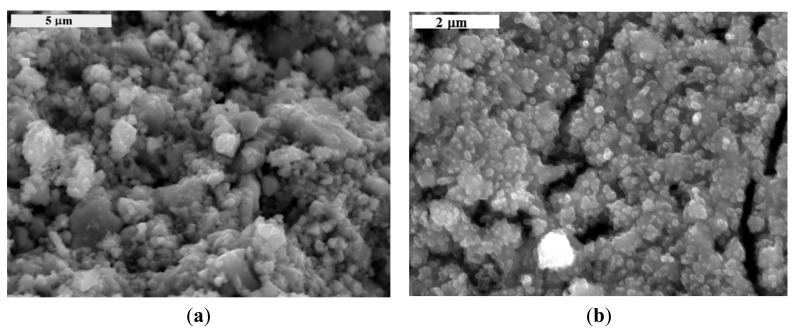
Surface microstructure of metal oxide layers: (**a**) SnO_2_; (**b**) WO_3_.

The determined average size of tin dioxide crystallites was 25.5 nm and that of tungsten trioxide was 24.8 nm. As shown in Figure 5a,b, the testing sensor materials, SnO_2_ and WO_3_, are the agglomerate of the crystallites. It was also found that there are few a narrow opening or crack in the tungsten trioxide layer.

**Figure 6.**
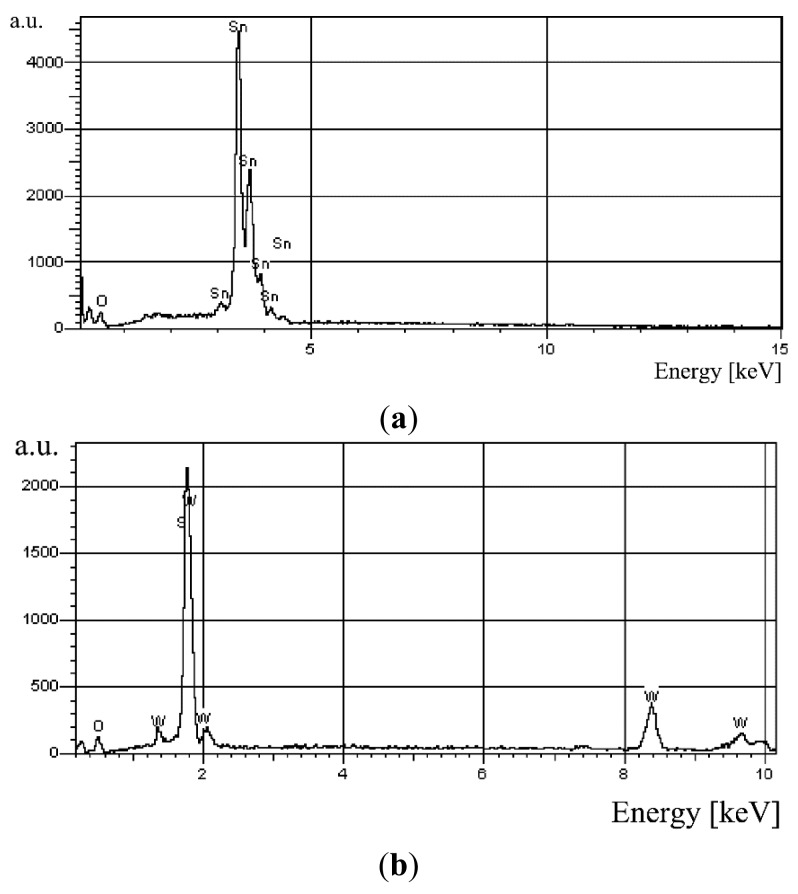
EDS spectrum of the layer: (**a**) SnO_2_; (**b**) WO_3_.

The testing of the chemical composition of the applied gas-sensitive materials carried out using an X-ray microprobe demonstrated that tin dioxide contained main elements, that is oxygen and tin. In Figure 6a, in the case of SnO_2_, no presence of a characteristic X-ray radiation peak of chloride ions (Cl^−^) which are a constituent of the precursor (SnCl_4_). On the other hand, in Figure 6b, the EDS analysis of the WO_3_ layer revealed that in the one, beside tungstic and oxide atoms, silicon was also present. The admixture of silicon in the WO_3_ layer is the result of the silicon presence in the precursor, that is tungstic-silicic acid H_8_[Si(W_2_O_7_)]_6_.

**Figure 7.**
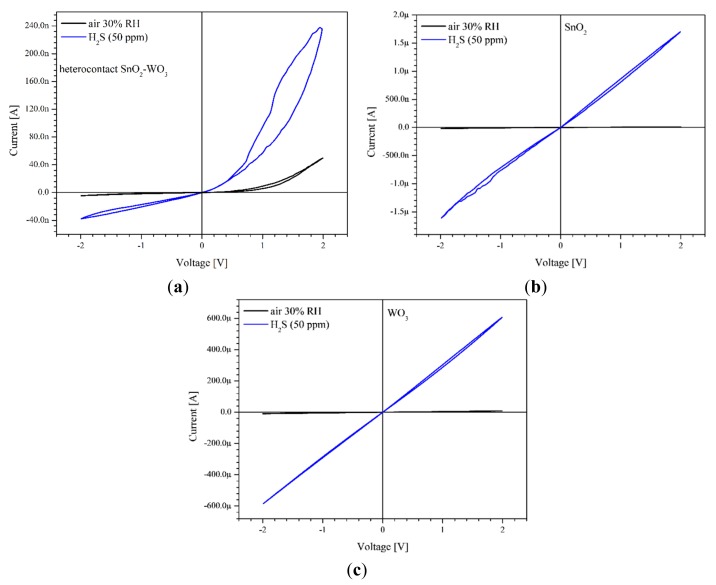
Current-voltage characteristics of sensors with: (**a**) SnO_2_|WO_3_ heterocontact; (**b**) SnO_2_ layer; (**c**) WO_3_ layer in the air atmosphere and air that containing 50 ppm of hydrogen sulphide. Relative humidity of air 30%; measurement temperature 300 °C.

The tested sensor layers were made from two semiconductive metal oxides *n*-type, undoped, that formed a heterocontact SnO_2_|WO_3_. Characterization by cyclic voltammetry of heterocontacts allowed us to determine the current-voltage characteristics (I-V) under an atmosphere of dry air with a different composition. The current-voltage measurements revealed that the electric current flowing through sensor layers, which formed a heterocontact, was not a linear function of the polarization voltage (Figure 7). This means that the formed heterocontact of *n-n* type has the properties of a rectifying connection (Schottky's contact). On the other hand, the current-voltage characteristics of sensors with layers built only from single gas-sensitive materials are linear (Figure 7b,c).

The presence of a reducing gas (hydrogen sulphide) very clearly affects the current value but does not change the nature of the current-voltage characteristics (Figure 7).

In the hydrogen sulphide atmosphere, under the same working point conditions (T = const, V_pol_ = const), the electric current increases, independent of polarization direction. In the case of all three sensors being tested the level changes depended on the gas concentration and temperature. In the case of the sensor with a heterocontact it also depended on the direction and the value of polarization voltage. When the potential difference was 2 V, and the heterocontact was polarized in the reverse direction, then an almost ten-fold rise in the current intensity in relation to the value determined in air was noticed. In turn, when the heterocontact was polarized in the forward direction, this rise was almost five-fold. The results revealed that the polarization of the heterocontact formed by direct contact of WO_3_ and SnO_2_ layers is present in the direction:

- the reverse direction when: (+) WO_3_|SnO_2_ (−),
- the forward direction when: (−) WO_3_|SnO_2_ (+).

The rectifying nature of the formed heterocontacts was the result of the use the semiconductive oxide materials with different value of:

- the work function Φ,
- the width of the band gap energy *E_g_*,
- the concentration of carriers in the conduction band *n_c_* [13,14].

The work function of these materials determined using the Kelvin probe from KP Technology (Wick, Scotland) is Φ*SnO*_2_ = 5.3 eV, Φ*WO*_3_ = 5.05 eV [12], respectively, and the width of the energy gap *E_g_* for SnO_2_ totals 3.5 eV [15], and for WO_3_ is 2.8 eV [16]. On the other hand, the concentration of electrons in tin dioxide ranges from 10^21^ to 10^23^ m^3^ [17] and in tungsten trioxide from 10^21^ to 10^24^ m^3^ [18]. As in the typical resistive gas sensors, so in the case of heterojunction sensors, temperature has a significant impact on their electrical parameters. For this reason a characterisation of heterocontacts in function of temperature was made. The characterisation was made within the range of 150 °C to 750 °C, in the atmosphere containing 50 ppm of hydrogen sulphide.

**Figure 8.**
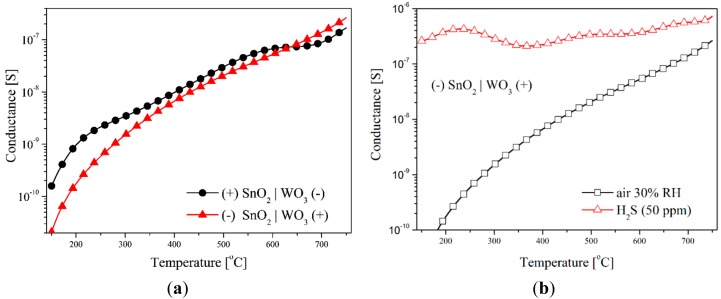
Temperature characteristics of TS-conductance of sensors with a heterocontact at various polarization directions in the atmosphere of: (**a**) synthetic air; (**b**) air containing 50 ppm of hydrogen sulphide.

The testing of the sensors with the SnO_2_|WO_3_ heterocontact with the TSC method, revealed that temperature changes in conductance depends on the direction of polarization, independently of the composition of the gas atmosphere (Figure 8). During this testing, the value of the polarization potential was ±2 V. The impact of the polarization direction was clearly visible within the low temperature range in synthetic air (Figure 8a). On the other hand, in the atmosphere that contained hydrogen sulphide this impact was visible within the range of average and high temperature values (Figure 8b). This effect can be a result of the rise in the concentration of electric charge carriers as well as of the variations of the work function and the width of the band gap energy.

**Figure 9.**
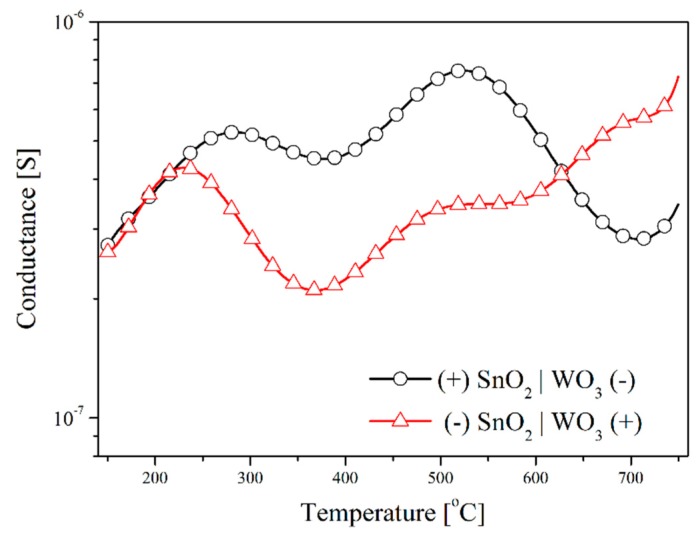
Temperature characteristics of TS-conductance in the atmosphere of 50 ppm H_2_S of sensor with a SnO_2_|WO_3_ heterocontact in function of polarization direction.

The addition to the synthetic air of hydrogen sulphide (50 ppm) causes a huge rise in the conductance of the layer with a heterocontact, especially in the low temperature range. In this atmosphere the conductance value changes insignificantly in the polarization direction but it behaves non-monotonically (Figure 9).

The observed non-monotonic changes in conductance as a function of temperature may be due to two reasons. Firstly, due to the differences in the kinetics of the chemical processes occurring on the surface of the applied sensor materials, and secondly, due to the difference in physical-chemical processes occurring on the surface and in the volume of both materials due to the temperature increase. Depending on the temperature, both in the volume and on the surface of the gas-sensitive materials occur different physical and chemical processes (Table 1) associated with the reaction of oxygen and water on their surface [19]. On the basis of the detailed analysis of the test results and literature data, the authors claim that the negatively polarized type of the material have a decisive influence on temperature changes in conductance. At temperatures above 150 °C the transformation (1) begins, and therefore the catalytic oxidation of hydrogen sulphide begins (2) [20]. Maximum conductance depends on the direction of polarization, which may result from the different affinity of the sensor materials to hydrogen sulphide. It is well known that WO_3_ is a better gas-sensitive material to detect hydrogen sulphide than tin dioxide [21]. When the maximum value is reached, conductance decreases, which may be due to the depletion of one of the reactants (2), *i.e.*, oxygen ions. These ions are formed as a result of oxygen chemisorption on surface oxygen vacancies. Above 400 °C, bulk oxygen vacancies may diffuse to the surface of WO_3_ grains, becoming new reaction centres for oxygen adsorption and its further reaction with H_2_S [22]. As a result, a further increase in conductance above 400 °C is observed (Figure 9). This is a well-known phenomenon. The formation of chemical bonds between gas molecules and the metal oxides surface depends on the presence of non-saturated bonds on the surface of such materials. Therefore, the quantity of the chemisorbed particles increases along with an increase in the concentration of the surface defects [23]. The increase in conductance above 550 °C (the reverse direction) may be caused by the thermal decomposition of tin dioxide (5) [24]. The drop in conductance for polarization in the forward direction observed above this temperature, may be due to two reasons, *i.e.*, reduction in speed of oxidation of hydrogen sulphide on the surface of tungsten trioxide material and the desorption of ions (4). The increase in conductance that occurs above 700 °C may be a result of heat dissociation and WO_3_ sublimation (6) [25].

**Figure 10.**
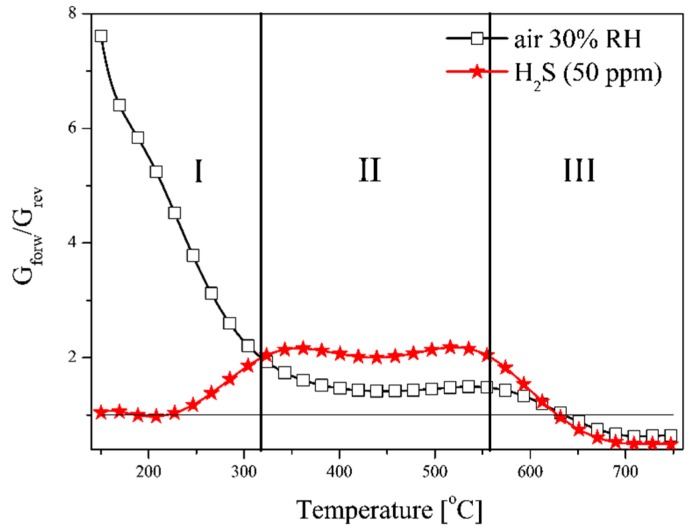
The temperature characteristics of the ratio of conductance as determined at various polarization directions in function of the composition of gaseous atmosphere.

The differences in the temperature characteristics of TS-conductance of the sensors being tested depending on polarization directions and the atmosphere composition are clearly visible on the characteristic curves in Figure 10, on which the dependence of the ratio of the conductance in the forward direction (G_forw_) to that in the reverse direction (G_rev_) was presented.

As the analysis of experimental data in Figure 10 clearly shows it is possible to differentiate three areas of conductance changes. In the air this ratio very strongly decreases below 330 °C. Taking into account the fact that regardless of the polarization direction, the kinetics of surface reaction occurring only with oxygen on the materials applied is similar, the observed change is caused by the change in the width of depleted layer of SnO_2_-WO_3_ heterocontact. The width of this layer can be described by the following Equation (3) [14]:

(3) $$W_{\left(\text{depletion width}\right)}=\sqrt{\frac{2{ɛ}_{s}}{qN_{D}}\left(V_{bi}-V-\frac{kT}{q}\right)}$$

where: ε_s_—semiconductor permittivity, *N_D_*—donor concentration, *V_bi_*—built-in potential, *V*—voltage, *q*—magnitude of electronic charge, *T*—temperature.

At temperatures above 250 °C, water begins to decrease and therefore the effect of polarization on the conductance value of the tested sensors in the H_2_S atmosphere begins to be observed [20,29]. In zone II, in the temperature range from 350 °C to 550 °C, there is a larger influence of the polarization direction of the sensor in the atmosphere of hydrogen sulphide than in the air. This may be due to higher sensitivity of WO_3_ in hydrogen sulphide than in tin dioxide [21]. At temperatures above 600 °C the polarization direction and the atmospheric composition have a comparable influence on the value of the conductance of heterojunction sensors, but the conductance of the sensors polarized in the reverse direction is higher than of those polarized in the forward direction (at ambient temperature). This is due to the predominant increase in the conductance of tin dioxide caused by its thermal dissociation (5). Reduction of conductance in the forward direction to conductance in the reverse direction in the air is caused by an increase of conductance in both materials and its decrease with increasing temperature.

**Figure 11.**
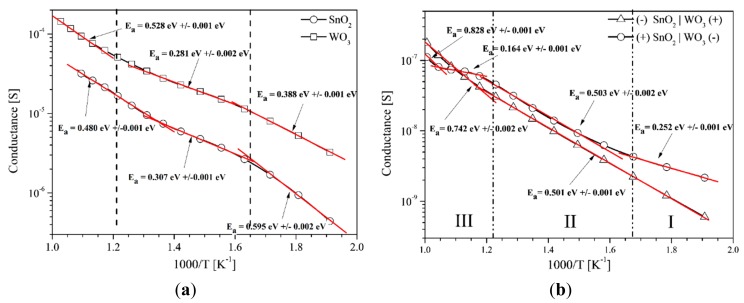
Change in conductance and activation energy of: (**a**) tin dioxide and tungsten trioxide layers; (**b**) SnO_2_|WO_3_ heterocontact in function of the inverse of temperature in synthetic air. The reverse direction: (+)WO_3_|SnO_2_(−); the forward direction: (−)WO_3_|SnO_2_(+).

In order to clarify the mechanism of phenomena occurring in the sensor with a heterocontact being designed, its parameters were compared to those of a sensor the gas-sensitive layer of which was made from one type of metal oxides only (Figure 11).

The conductance of sensors with a single-phase layer does not depend on the polarization direction and is decidedly higher than that of sensors with a heterocontact in the atmospheric air. The values of the activation energy of individual sensors, within the same temperature ranges, are also different. Lack of influence of the polarization direction on the sensors containing only one layer is due to their symmetrical structure, which can be described as Au/SnO_2_/Au. It also means that in the sensors with SnO_2_ and WO_3_, gold electrodes form an Ohmic contact, which is also confirmed by the result of the current-voltage characteristics presented in Figure 7b,c.

The value of the activation energy of the electric conductivity, presented as clearly dependent on the polarization direction, which indicates the impact of the heterocontact on the process of transporting the electric charge in the test sensors (Figure 11b). This is particularly clearly visible when the sensor is polarized in the reverse direction. In the sensors polarized in the forward direction (−)WO_3_|SnO_2_(+) four ranges of changes in the value of the activation energy of electrical charge transport may be distinguished. In zone I the value of activation energy equals the difference of work function of the sensor materials used. Therefore, in this temperature range, chemisorption of oxygen, which occurs on the surface of the gas-sensitive materials has a small impact on the transport of the electric charge. In the second zone the activation energy value is the same as the value of polarization in the reverse direction (+)WO_3_|SnO_2_(−). In this polarization direction, the value of the activation energy is constant and is almost twice larger than the difference in the work function of tin dioxide and tungsten trioxide (0.25 eV). This is due to the presence of the barrier at the border of the layers present and the diffusion of oxygen vacancies in WO_3_ described in Table 1. At temperatures above 550 °C (zone III), in the case of sensors polarized in both directions, the impact of thermal dissociation of carbon and WO_3_ sublimation is observed. Both processes require high activation energy.

**Figure 12.**
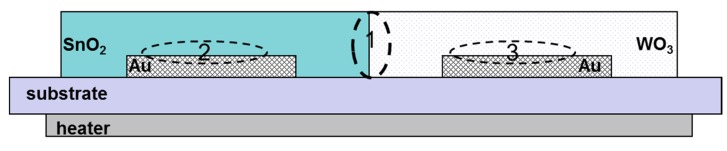
Schematic diagram of the cross-section of the sensor with a heterocontact.

The sensor materials used, as shown by the tests performed using a scanning electron microscope (SEM), are polycrystalline materials. However, they are commonly used in chemical resistive gas sensors due to their large specific surface area, which allows the effective surface chemical reactions when the sensors work [22]. The efficiency of these reactions, which are typical of the heterogeneous catalysis, determines the performance characteristics of these sensors [13,30]. For the analysis of the phenomena occurring in these sensors, the monocrystalline semiconductor theory is commonly used, being aware of its limitations [31–33]. In our study, the analysis of the phenomena occurring in the described sensor with heterocontact also applied this theory. Within the area of the designed sensor three inter-phase boundaries can be distinguished (Figure 12). The SnO_2_–WO_3_ heterocontact is one inter-phase boundary (No. 1), and the metal/metal oxide semiconductor (No. 2) Au/SnO_2_, (No. 3) Au/WO_3_ are the two remaining ones.

Each of these phase boundaries as well as the properties of oxides determine the final parameters of the investigated sensor. However, during the analysis of phenomena occurring in the sensors with heterocontact, it was adopted that only the phase boundary between the oxides has an essential impact on the parameters of the sensor. The assumption was adopted based on the reference data and our own experience gained during the research work on chemical gas sensors [34].

**Figure 13.**
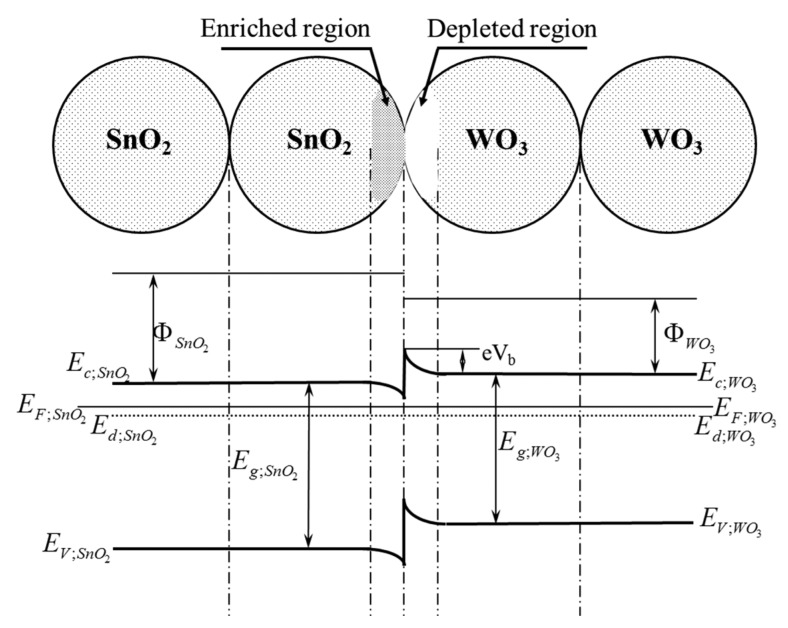
Schematic diagram of the contact of the grains of tin dioxide and tungsten trioxide as well as the band structure of these contacts in vacuum.

In vacuum, in relation to the foregoing assumption, only one potential barrier will be formed in the tested sensor containing a heterocontact, at the boundary of the grains of tin dioxide having a direct contact to those of tungsten trioxide (Figure 13).

**Figure 14.**
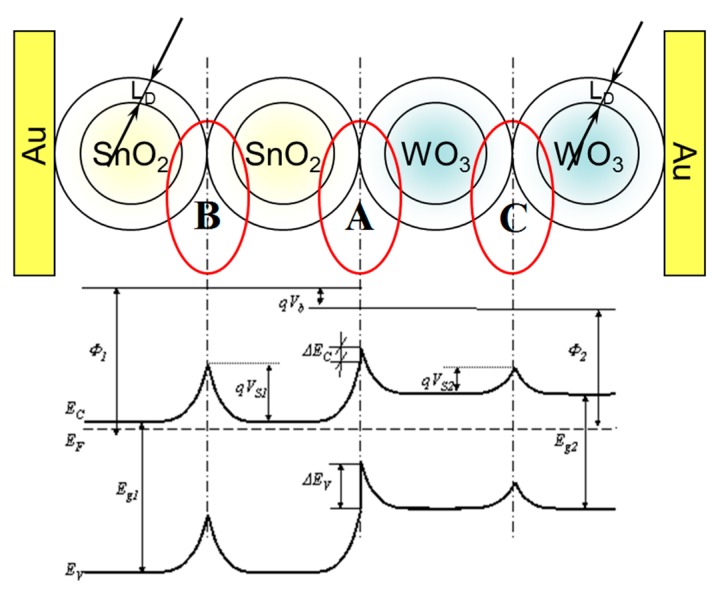
Schematic diagram of the contacts between the grains of tin dioxide and tungsten oxide as well as the band structure of the contacts formed in the ambient atmosphere.

This potential barrier (A) is also present in the ambient atmosphere, however, its height is different than in vacuum. Besides, in the ambient atmosphere, two potential barriers of a different height are additionally created (B; C) as a result of the interference of sensor materials with oxygen what was shown in Figure 14.

**Figure 15.**
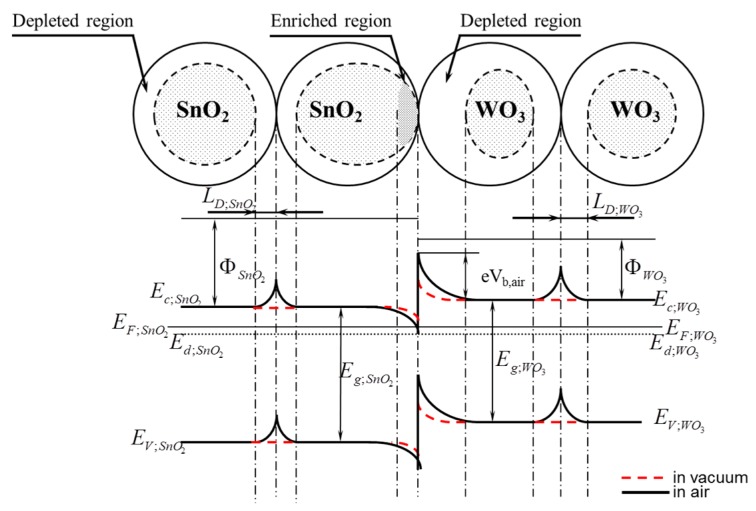
Schematic diagram of the contact of the grains of tin dioxide and tungsten trioxide as well as the band structure of these contacts in vacuum and the atmosphere that contains oxygen.

The band bending observed in the surface-based area is commonly known in the band model of semiconductive materials. One of the causes of this phenomenon is, among others, native surface states [14]. The band bending in oxide semiconductors is a result of the removal of anions from the lattice sites, *i.e.*, oxygen ions [35], whereas the root cause of the band bending on the surface of semiconductive oxide materials is the oxygen chemisorption (oxidative gas) or hydrogen chemisorption (reducing gas). The chemisorption process of these gases is always related to the exchange of electron concentration. For this reason, if the heterocontact finds itself in the atmosphere that contains oxygen (synthetic air), then as a result of the oxygen chemisorption a depleted layer will be formed (*L_D_*—effective Debye's length) on the surface of all grains, *i.e.*, on the surface of both tin dioxide and tungsten trioxide (Figure 15). In such a case there will be three potential barriers in the sensor being tested, having a various height and a various width of the surface charge density.

In the atmospheric air the heights of potential barriers (B; C) and the widths of depleted layers *L_D_* depend on the kinetics of the oxygen chemisorption process, and during the determination of gases, also on the kinetics of oxidation and reduction processes of gases having reducing or oxidizing properties, respectively [32]. For this reason the conductance of metal oxides of semiconductive properties in the synthetic air can be expressed by the Equation (4), and in the air containing the gas being determined, by the dependence from the Equation (5):

(4) $$G_{a}=G_{o}\text{exp}(-eV_{a}/k_{B}T)$$

(5) $$G_{gas}=G_{o}exp(-eV_{gas}/k_{B}T)$$

where: *G_gas_*—conductance in the gaseous atmosphere containing the gas being determined, *G_a_*—conductance in the reference atmosphere, *k_B_*—Stefan-Boltzmann constant, *T*—absolute temperature, *e*—value of the elementary charge, *i.e.*, 1.602 × 10^−19^ C, *V_gas_*—potential barrier in the gaseous atmosphere, *V_a_*—potential barrier in the synthetic air.

As mentioned before, since the measurements made using the Kelvin probe from KP Technology demonstrated that the value of the work function from tin dioxide was 5.30 eV, and it was higher than that from tungsten trioxide that totals 5.05 eV, therefore in the area of the heterocontact, on the side of WO_3_, a depleted layer is formed, and on the side of SnO_2_, an enriched layer is formed. In such a case the edges of the conduction band and those of the valence band in the heterocontact area change its potential energy by the total value expressed by the Equation (6):

(6) $$\Delta E=e(V_{SnO_{2}}+V_{WO_{3}})=\frac{e^{2}}{2{ɛ}_{r}}(n_{SnO_{2}}L_{SnO_{2}}^{2}+n_{WO_{3}}L_{WO_{3}}^{2})$$

where: *V*_*SnO*_2__—potential barrier height on the tin dioxide side, *V*_*WO*_3__—potential barrier height on the tungsten trioxide side, *n*_*SnO*_2__—concentration of electrons in tin dioxide, *n*_*WO*_3__—concentration of electrons in tungsten trioxide, *L*_*SnO*_2__—width of the enriched layer in tin dioxide, *L*_*WO*_3__—width of the depleted layer in tungsten trioxide, *ε_r_*—relative permittivity of the material.

The width of the depleted layer in the boundary layer of tungsten trioxide will be different than in tin dioxide since its width depends on the concentration of electrons in the given oxide and on temperature expressed by the Equation (7) [14]:

(7) $$L_{D}=\sqrt{\frac{{ɛ}_{0}{ɛ}_{r}k_{B}T}{e^{2}n_{b}}}$$

where: ε_0_—vacuum permittivity, ε*_r_*—relative permittivity, *n_b_*—concentration of electrons.

Since the gas-sensitive layer with a heterocontact is formed from two chemically different materials, therefore the heights of potential barriers formed between the grains of tin dioxide and those of tungsten trioxide are not equal to each other and do not change identically in function of temperature and the composition of atmosphere. As mentioned previously, the concentration of electrons in tin dioxide is different than in tungsten trioxide [30], therefore the width of the depleted layer formed in the WO_3_ layer will be greater than in the SnO_2_ layer (Equation (8)):

(8) $$L_{D(SnO_{2})}<L_{D(WO_{3})}$$

The formation of the depleted layer on the surface of grains will also affect the height of the potential barrier being formed at the phase boundary; tin dioxide—tungsten trioxide. It is commonly adopted that the height of the potential barrier (A) formed due to a direct contact to one another of two semiconductors of various electric parameters, as shown in Figures 14 and 15 depends, first of all, on the electric parameters of contacting semiconductive materials, *i.e.*, work function, energy gap width, density of carriers. Taking into account that a heterocontact is formed by two oxide materials the resistance of which changes not only under the influence of temperature, but also under that of the composition of the ambient atmosphere, therefore the height of the potential barrier (A) should also depend on the composition of ambient atmosphere. Unfortunately, its direct impact on the height of the barrier is difficult to assess since the kinetics of the chemical adsorption process on the surface of these oxides depends on the type and concentration of electrons. In turn, the conductance of such a gas-sensitive layer will also depend on the conductance of materials as well as on that of a heterocontact.

**Figure 16.**
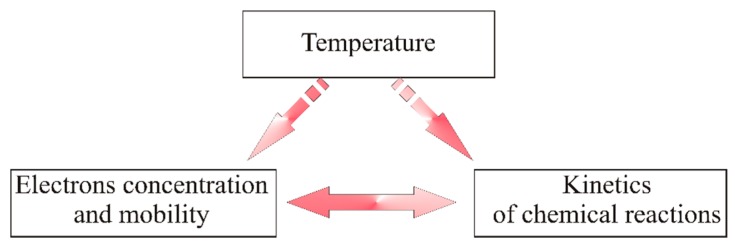
Impact of temperature on the physicochemical parameters of a sensor material.

Besides, the impact of the temperature on the value of the conductance of the gas-sensitive layer with a heterocontact is difficult to assess since it can be both direct and indirect. As illustrated in Figure 16, a change in the temperature of a material having semiconductive properties is a direct factor generating a change in the concentration and mobility of electric charge carriers, and it affects the kinetics of chemical processes.

The impact of the composition of a gaseous atmosphere on the conductance of the gas-sensitive layer with a heterocontact is the result of physicochemical processes occurring on the surface of oxide semiconductive materials, in their volume (e.g., at the boundary of grains, at the contact interfaces of grains, in the areas being close to electrodes) [34] and in the connection area. The physical processes occurring in the connection area depend on the electric parameters of materials, the temperature and the kinetics of chemical processes occurring in the area of a heterocontact. On the other hand, the physicochemical processes occurring on the surface of semiconductive oxides depend on: kinetics of the oxygen chemisorption, type and composition of the oxide material, chemical nature of the gas being determined.

Due to a relatively small width of the SnO_2_|WO_3_ contact (*ca.* 10 μm) in comparison to the spacing between the electrodes (500 μm), it can be assumed that the presence of the gas being determined does not affect the parameters of the heterocontact. The analysis of the conductivity variation in function of the inverse of temperature at various polarization directions in air, presented in Figure 11a,b, indicates that the connection area essentially affects the conductance of the whole gas-sensitive layer. The conductance of such a gas-sensitive layer is not only decidedly lower than that of individual oxides. However, the nature of temperature variation of this parameter is different.

In the atmosphere containing the gases being determined, oxidation processes occur with the use of chemisorbed oxygen ions on the surface of oxide semiconductive materials. These processes can be presented by the Equation (9) as the dissociation reaction of hydrogen atoms from the central atom:

(9) $$H_{2}S+3O_{ads}^{-}\overset{T,Me_{x}O_{y}}{⟷}SO_{2}+H_{2}O+3e^{\prime }$$

As a result of the abovementioned reactions, products of a various number of oxygen bound with the central atom of the substrate are obtained. The dissociation process of hydrogen atoms and afterwards, the association process of oxygen atoms is particularly visible during the oxidation reaction (determination) of organic compounds.

Irrespective of the type of the process occurring during the detection of various gases on the surface of a semiconductive oxide the concentration of charge carriers increases and the height and width of the spatial charge area decreases. On the other hand, the kinetics of both the oxygen chemisorption process and that of the oxidation process of gases being determined depends on the type and composition of the sensor material and on the chemical nature of the gas being determined. Hence it is difficult to foresee how the height of the potential barrier and the width of the spatial charge area will change in the connection area and by what sensitivity the given system will be characterized.

An insignificant impact of the polarization direction and that of temperature on the electric parameters of the heterocontact being tested in the presence of hydrogen sulphide can be a result of a very high constant reaction rate generating a high increase in the concentration of both the sensor materials. Then, in compliance with the dependence presented in Equation (7), both the height of the potential barrier and the width of the spatial charge area will decrease.

## 4. Conclusions

The paper presents the test results of a sensor in which the sensor layer was built from two different types of sensor materials. Tin dioxide was printed on one electrode and tungsten trioxide was printed on another one. The testing demonstrated that the intensity of electric current flowing through such a sensor layer was not a linear function of the polarization voltage, therefore the formed contact type *n-n* has the properties of a rectifying connection (Schottky's contact). The rectifying nature of the heterocontact is a result of the application for the construction of a gas-sensitive layer of two semiconductive materials having different properties. The presence of the heterocontact resulted in an essential rise in the resistance of the sensor layer in comparison to that of sensors containing a gas-sensitive layer built from one type of oxide. In the area of the gas-sensitive layer of the sensor being tested with a heterocontact three inter-phase boundaries can be distinguished, that is two electrode/sensor material boundaries and one boundary between two SnO_2_/WO_3_ sensor materials applied. However, the authors assumed that the parameters of the sensor being tested are essentially affected only by the inter-phase SnO_2_/WO_3_ boundary since the current-voltage characteristic curves of the sensors containing a sensor layer built from only one type of sensor material are linear. Consequently, in the sensor being tested, three areas can be distinguished, differing in the width of the depleted layer, thus the areas in which the rate of the occurring chemical processes is different. If the rate of those processes is different, then it affects, in a different manner, the conductance of the individual areas of the sensor material, accordingly also the resultant output signal of the sensor.

As demonstrated by the test results, the impact of the heterocontact strongly affects the parameters of the sensor, therefore, in the opinion of the authors of this article, the construction of sensors whose gas-sensitive layer contains a heterocontact allows an essential modification of the useful parameters of the sensors.

## Figures and Tables

**Table 1.** Physical and chemical changes of tin dioxide/tungsten trioxide due to adsorption of oxygen or water on its surface.

**Process No**.	**Temp. [°C]**	**Physical and Chemical Changes**	**References**
(1)	150	desorption of $O_{2}^{-}$ and transformation of $O_{2}^{-}$ → *O*^−^ begins	[26,27]
(2)	>150	3*O*^−^ + *H*_2_*S* → *H*_2_*O* + *SO*_2_ + 3*e*^−^	[20]
(3)	>400	diffusion of oxygen defects to the surface WO_3_ and formation of new adsorption centres	[22]
(4)	>520	*O*^−^ desorption	[26]
(5)	>550	thermal dissociation of SnO_2_ $SnO_{2}↔Sn_{Sn}^{x}+2V_{o}^{¨}+4e^{\prime }+O_{2}↑$	[24]
(6)	>700	sublimation of WO_3_	[25,28]
